# Is PML a Tumor Suppressor?

**DOI:** 10.3389/fonc.2013.00174

**Published:** 2013-07-09

**Authors:** Massimiliano Mazza, Pier Giuseppe Pelicci

**Affiliations:** ^1^Department of Experimental Oncology, European Institute of Oncology, Milan, Italy

**Keywords:** PML, tumor suppressor, cancer stem cells, cancer, cancer therapy

## Abstract

The role of the promyelocytic leukemia (PML) protein has been widely tested in many different contexts, as attested by the hundreds of papers present in the literature. In most of these studies, PML is regarded as a tumor suppressor, a notion on the whole accepted by the scientific community. In this review, we examine how the concept of tumor-suppressor gene has evolved until now and then systematically assess whether this assumption for PML is supported by unambiguous experimental evidence.

## Tumor Suppressor, an Evolving Concept

Despite more than 80 years of research on “characterizing properties,” the tumor-suppressor definition is still controversial. The existence of chromosomal inherited traits that negatively regulate tumor development was first postulated by Theodor Boveri (1), who suggested the existence of inhibitory chromosomes that keep cell proliferation in check and favor tumor development upon removal. Formal proof for this concept came from cell fusion experiments by Henry Harris, who showed that hybrids of normal rodent cells and cancer cells exhibited growth properties similar to normal cells (2).

Few years later, Alfred Knudson Jr. (3) proposed a genetic mechanism for hereditary retinoblastoma (RB) that has influenced cancer genetics until today. Knudson, on the basis of mathematical and statistical analyses, hypothesized that the disease was caused by two rate-limiting genetic lesions, one inherited and phenotypically silent, and one acquired somatically during life. His theory was then validated by cloning the *RB* gene, and it is commonly referred to as the “two hit hypothesis.” In his model, Knudson introduced a new class of cancer genes, the recessive anti-oncogenes, now known as tumor-suppressor genes (TSGs) (4–6). Since then, the two hit model has defined the experimental strategies for the identification of novel TSGs: (i) identification of genes involved in cancer-predisposition, where the first “lesion” is inherited and the second is acquired somatically; (ii) identification of genes inside genomic regions displaying loss-of-heterozygosity (LOH) in cancer cells; (iii) identification of chromosomal regions from normal cells that suppress the transformed phenotype. Those strategies are embedded in the concept of double mutation, emphasizing this aspect over gene function. For this reason many today consider two inactivating hits in cancer to be the only acceptable proof for the existence of a TSG.

In 1997, Haber and Harlow proposed a novel description of TSGs as “genes that sustain loss-of-function mutations in the development of cancer” (7), thus introducing a functional aspect in the definition. The same year, Kinzler and Vogelstein distinguished two functional classes of TSGs, namely, “gatekeepers” and “caretakers” (8). Gatekeeper genes are designated to control cell-cycle progression, e.g., adenomatous polyposis coli (*APC*), β-catenin (*CTNNB1*), and RB, whereas caretaker genes maintain genome integrity, e.g., breast cancer gene 1 (*BRCA1*), Bloom syndrome gene (*BLM*), and ataxia telangiectasia mutated gene (*ATM*). Caretaker mutations would lead to increased mutation rate, consequently increasing the probability that gatekeepers become inactivated. There are several studies on caretaker and gatekeeper genes in the literature and the examples that follow below have been intentionally chosen to reject the belief that two inactivating mutations are required to define a tumor suppressor.

*CDKN1B*/p27KIP1 is a member of the cyclin-dependent kinase inhibitor family and regulates cell-cycle progression. Two series of evidence suggest that *CDKN1B* behaves as a classical TSG: LOH at chromosome 12p13, encompassing the *CDKN1B* locus, is frequently found in breast, prostate, and ovarian cancers (9–14), and reduced CDKN1B protein is significantly associated with high-grade and high-stage disease in those tumors [reviewed in (14)]. Reverse genetic experiments in mouse models, however, suggest a different scenario: (i) *Cdkn1b*^−/−^ and *Cdkn1b*^±^ mice developed intestinal and lung adenomas at higher frequency than wild type mice, yet with similar latency and penetrance (15); (ii) tumors from *Cdkn1b*^±^ mice did not show mutations of the second *Cdkn1b* allele; (iii) the CDKN1B protein was still expressed at about 50% of the levels of wild type animals. In this case, as for other genes like *Dmtf1* (16, 17), *Fhit* (18, 19), and *Apc* (20), to mention a few, it is clear that the mutation of a single allele is sufficient to promote tumorigenesis, a phenomenon that is called haplo-insufficiency. Haplo-insufficient TSGs have been proposed to act by increasing the population of mutated cells available for further mutations [reviewed in (21)].

Haplo-insufficiency is not the only exception to the two hit model. For example, *ATM* missense mutations are more common than *ATM* null mutations in breast cancers (22, 23), and mice carrying a copy of a mutated *Atm* allele develop more tumors than mice heterozygous for a silenced *Atm* allele (*Atm*^±^) (24), suggesting the existence of mono-allelic, dominant negative mutations in tumor suppressors. Thus, in open contrast to the classical definition of TSGs, dominant negative and haplo-insufficient genes are able to give rise to tumors following a single mutation insult. Finally, genetic mutations are not the only mechanism of functional inactivation for TSGs. Analysis of *CDKN2A* (p16INK4) in primary tumors, for example, revealed few inactivating mutations, and *CDKN2A* CpG island hypermethylation in 20% of cases (25), suggesting that epigenetic silencing of TSGs can drive tumorigenesis as well.

Mounting evidence and exceptions to the two hit model suggest that this definition of TSGs is too restrictive and in need of revision. A TSG operative definition should probably focus more on functional aspects, clear support from reverse genetic experiments, and determination of gene/protein dosage for which the two hit model is the *extrema ratio*. According to this view, we would then define a TSG as a gene whose alteration is found in human tumors, causes a reduction of its function, favors tumorigenesis (as demonstrated by the introduction of the same modification in animal experimental models), and if the overexpression of its wild type form elicits anti-tumoral effects. Functional reduction can be due to a reduced level of expression and to mutational events, including dominant negative effects on the wild type allele. With this paradigm in mind we questioned whether promyelocytic leukemia (PML) fulfills all the above requirements to be considered a true TSG.

## PML Expression in Human Tumors

The first criteria we have examined to assess if PML is a tumor suppressor is the presence of genetic alterations or abnormal expression in human cancers.

About 95% of human acute promyelocytic leukemia (APL) cases harbor the oncogenic fusion gene *PML-RARA* as a result of the translocation t(15;17), whose breakpoints are located within the *PML* and the retinoic acid receptor α, *RARA* loci on chromosome 15 and 17, respectively. In principle, *PML* function in these cells is impaired by two concomitant events: *PML* haplo-insufficiency, for the presence of a single *WT PML* autosome and the expression of PML-RARA, which might interfere with the function of the WT PML protein. PML is involved in the regulation of many cellular functions and some of them, such as the activation of P53 by the DNA damage checkpoint response, rely on its localization and organization in subnuclear structures called PML-nuclear bodies (PML-NBs). PML-RARA functions as a dominant negative factor in the process of PML-NB assembly, causing a microspeckled distribution of PML. In this context, treatment with retinoic acid and arsenic, two agents that induce disease-remission in APL patients, leads to PML-RARA degradation, cell differentiation, re-assembly of PML-NBs, and tumor regression. These “therapeutic” effects establish a correlation between PML-NB assembly and tumor suppression, yet formal evidence that PML-NBs *per se* are responsible for tumor suppression is still missing. Indeed, it cannot be excluded that APL regression is due, exclusively, to other missing PML-RARA-associated functions after its degradation. Notably, expression of X-RARA chimera, which does not interfere with PML-NB assembly (P50-RARA, GCN4-RARA), and where X represents a coiled-coil domain mediating RARA homodimerization, maintains transforming potential, and recapitulates the main biological properties of PML–RARA (26, 27). However, the expression of X-RARA fusion proteins in mice, like P50-RARA or GCN4-RARA, is not sufficient to drive tumorigenesis as PML-RARA or the CC^PML^-RARA protein, where the RARA is fused to the coiled-coil region of PML, do (26). *Pml*^−/−^ mice expressing *P50-Rara* do not show increased incidence or acceleration of leukemia onset. This suggests that loss of *Pml* cannot complement *P50-Rara* in restoring the leukemogenic potential to a level comparable to that of PML-RARA, and that the *PML-RARA* oncogene does not simply interfere with *Pml* and *Rara* functions but has additional activities that cannot be recapitulated by separating the two components in this way (27). In contrast with this observation, *Pml^−/−^ Pml-Rara* transgenic-mice show increased incidence and acceleration of leukemia onset (28). Therefore is not clear if PML-NBs really exert tumor suppressive functions or not.

Immunohistochemical analysis of PML expression in human tumors of different histologic origins shows that PML expression-levels are reduced in a considerable number of cases, as compared to the corresponding normal tissues. PML expression is absent in 49% of central nervous system (CNS) tumors (in 100% of medulloblastomas and over 90% of oligodendroglial tumors), 17% of colon adenocarcinomas, 21% of lung tumors, 27% of prostate adenocarcinomas, 31% of breast adenocarcinomas, 49% of germ cell tumors, and 68% of non-Hodgkin’s lymphomas (in 83% of diffuse large-cell lymphomas and 77% of follicular lymphomas) (29). In addition, PML low-expression correlates with bad prognosis and high-grade tumors for breast adenocarcinomas and prostate carcinomas (29). PML is phosphorylated by the extracellular signal-regulated kinase ERK2 which facilitates the recruitment of the peptidyl-prolyl cis/trans isomerase PIN1 and the following degradation of PML by the proteasome (30, 31). Given the frequent activation of ERK2 in several types of tumors, due to the sustained action of paracrine growth factors or mutations, the ERK2/PML axis may partly explain why PML expression is low in a considerable fraction of human tumors. However, mutational analysis of the *PML* gene in 132 samples from different primary tumors and human cell lines showed that *PML* is rarely mutated or subjected to LOH mutations. Moreover, the few *PML* mutations detected did not correlate with PML protein-loss, suggesting that mutation is not the main mechanism of PML inactivation in the tumor types analyzed (29).

Triple negative breast cancer (TNBC) is a noticeable exception to the common theme of PML loss in tumors. PML expression is strongly associated with TNBC and basal high tumor-grade breast cancers, which are among the most undifferentiated and untreatable breast cancers (32). Here, high levels of PML expression correlate with early tumor recurrence, a signature of poor prognosis, and mutations of the tumor-suppressor *P53* (33). *In vitro* experiments performed on MCF10A cells overexpressing PML show increased survival to anoikis *via* the regulation of PPAR-α and fatty acid oxidation, suggesting a crucial role for PML in the regulation of cell metabolism (33).

Chronic myeloid leukemia (CML) is also an exception. Indeed, high levels of PML expression correlate with bad prognosis in CML, and its function is critical to maintain leukemic initiating cells (34). PML degradation induced by As_2_O_3_ (arsenic trioxide) treatment allows exit from quiescence and exhaustion of cancer stem cells (CSCs) in a murine model of the disease (34). This parallels the role of PML in hematopoietic stem cells (HSCs) where it is highly expressed and controls HSC self-renewal and symmetric division through its regulation of mTOR and PPAR-δ signaling (fatty acid oxidation) (34). Additional proof of the general role that PML plays in stem cell homeostasis comes from studies on the nervous system. In mice PML expression is restricted to neural progenitor cells (NPCs) in the developing neocortex where PML regulates NPC proliferation and differentiation (35).

In summary, analysis of PML expression in human tumors reveals two distinct situations: (i) reduced/loss-of expression in different tumor types, most frequently in CNS tumors, which suggests that *PML* may be a tumor suppressor; (ii) high expression in CML and TNBC tumors, which, on the contrary, rely on PML expression to, respectively, maintain unlimited self-renewal and survive under metabolic stressing conditions. A controversial picture emerges from all these observations, indicating that the role of PML in human malignancies is context dependent.

## *Pml* Loss is Not a General Tumor-Promoting Event

*Pml*^−/−^ mice are fertile, with birth rates in line with the expected Mendelian frequency, and without gross phenotypic differences compared to *Pml*^±^ or *Pml*^+/+^ littermates (36). However, *Pml*^−/−^ mice are leukopenic, show an increased susceptibility to infections, a reduction of both granulocytes and monocytes, and have an impaired capacity for terminal maturation of myeloid cells in response to retinoic acid (36). *Pml*^−/−^ mouse embryonic fibroblasts (MEFs) show an increased rate of proliferation as compared to *Pml*^±^ or *Pml*^+/+^ MEFs without the hallmarks of transformation (36). Notably, *Pml*^−/−^ mice do not develop spontaneous tumors at higher frequency than *WT* syngenic mice.

Experiments of cooperation between carcinogens/oncogenes and the concomitant inactivation of *Pml* show a more complicated picture. Cooperation between *Pml-Rara* and *Pml* inactivation was assessed in a murine model of APL expressing the *Pml-Rara* under the control of the human cathepsin G promoter (28). In this case, the leukemia-free survival (LFS) of *hCG-Pml-Rara*^±Pml^^−/−^ and *hCG-Pml-Rara*^±Pml^^±^ mice was significantly reduced as compared to *hCG-Pml-Rara*^±Pml^^+/+^ mice [mean LFS ± SD, respectively: 434.4 ± 30.6 days (*p* < 0.0001), 498.9 ± 31.3 days (*p* = 0.003), 686.4 ± 35.5 days] showing a clear acceleration of leukemia onset. Moreover, the incidence of leukemia was significantly increased in *Pml* absence or haplo-insufficiency (*p* < 0.001 for both) (28). However, these data were not confirmed in a second independent model system where *WT* and *Pml*^−/−^ cells transduced with a retroviral vector expressing *Pml-Rara* were compared for their ability to generate leukemia upon transplantation into irradiated recipients. In contrast to the previous study, leukemias developed significantly faster in a *WT* background (*p* < 0.05) (37). Since the two experimental settings were different it is difficult to draw a definitive conclusion about the role of *Pml* in *Pml-Rara* – driven APL.

*Pml* cooperation has also been assessed in a murine model of lung tumorigenesis driven by the oncogene *K-Ras^G12D^*, which induces non-small cell lung carcinomas (NSCLC) in mice (38). In this model, *Pml*^−/−^ mice showed an increased tumor burden compared to *Pml*^+/+^ control animals, assessed as the number of lung carcinomas/mouse after 8 weeks of continuous expression of the *K-Ras^G12D^* oncogene (*p* < 0.05) (39).

In a murine model of kidney tumor driven by the haplo-insufficiency of the tuberous sclerosis protein 2 (TSC2), the picture, again, is different. *Tsc2* heterozygosity leads to high MTORC1 activity, to the development of kidney cysts and, after a long latency, to carcinomas (40, 41). Kidney tumor initiation, assessed by the number of cysts and small carcinomas/kidney, is not affected in *Pml^−/−^ Tsc2*^±^ mice. However, tumors from these mice were more vascularized, showed higher proliferation and a more aggressive histological profile (42) suggesting changes in tumor progression rather than tumor initiation.

*Pml*^−/−^ mice, challenged with 7,12-dimethylbenz(α)anthracene (DMBA) and 12-*O*-tetradecanoylphorbol-13-acetate (TPA) two-stage skin carcinogenesis, showed an increased number of papillomas indicative of benign tumor initiation but only a mild increase in the frequency of tumors undergoing malignant transformation (1.8 *Pml*^+/+^ vs. 2.3% *Pml*^−/−^) (36). Finally, *Pml* haplo-insufficiency or complete inactivation does not affect frequency, latency, and size of breast tumors in the MMTV/neu murine model of mammary tumorigenesis (28).

Thus, deletion of *Pml* does not lead to transformation *per se* but can favor tumorigenesis in some specific instances. Indeed, far from providing a coherent and unifying picture for the role of *PML* in tumorigenesis, these observations suggest that *PML* contribution depends on the specific background in which its inactivation is achieved.

## PML Functions and Tumor Suppressive Pathways

Promyelocytic leukemia is a pleiotropic protein placed at the crossroad of many regulatory pathways. DNA damage and repair, anti-viral response, metabolic adaptation, induction of apoptosis/senescence, and telomere maintenance are some of the important processes which regulate and are, in turn, regulated by PML. Aim of this section is to examine the main tumor suppressive pathways and factors that are engaged by PML.

### PML, cell death, and senescence

Promyelocytic leukemia modulates the activity and expression of the known tumor-suppressor P53. Indeed, *Pml*^−/−^ cells express less P53 than *WT* controls, both under steady-state conditions and upon γ-irradiation (43, 44). PML interacts directly with the DNA binding domain of the tumor-suppressor P53, colocalizes with P53 in the PML-NBs and acts as a P53 transcriptional coactivator (45–47). It activates P53 through multiple mechanisms: increasing its acetylation and phosphorylation (44, 46, 48–50), binding and inhibiting the main negative regulator of P53, the mouse double minute 2 homolog, MDM2 (51–53), and promoting P53 deubiquitination through the herpes-virus-associated ubiquitin-specific protease, HAUSP (54, 55). In turn, activated P53 can directly induce PML expression generating a positive feedback loop (56). Biologically, the influence of PML on the P53 pathway may result in the regulation of stress-induced apoptosis or senescence on a cell type-dependent basis. All these features are coherent with a tumor suppressive role of PML exerted *via* P53.

Promyelocytic leukemia function in apoptosis is not limited to the regulation of P53 or to the P53-dependent apoptotic pathway. Indeed, both murine *Pml*^−/−^ cells and *PML* siRNA interfered human cells are defective for the induction of apoptosis triggered by several factors, like type I and type II interferons (IFN), FAS, tumor necrosis factor, ceramides, ionizing radiations (57, 58), and transforming growth factor (TGF)-β, which induce apoptosis (59).

How can a single protein being involved in the regulation of apoptosis from so many different stimuli? PML has been shown to interact with the phosphatase PP2A and to modulate its activity on specific targets. *Via* PP2A PML can modify the sensitivity to pro-survival and pro-apoptotic cues in at least two ways: (i) recruiting PP2A phosphatase to nuclear PML-NBs, thereby de-phosphorylating and inactivating AKT and its pro-survival functions (60); (ii) recruiting PP2A to the inositol 1,4,5-trisphosphate receptor, IP3R-3, to modulate Ca^2+^ release and storage at the endosplasmic reticulum (ER). The physical structures where both this regulation is achieved and cytoplasmic PML is localized are called mitochondria-associated membranes (MAMs). Indeed, sensitivity to apoptosis is dependent on the ability of cells to transfer Ca^2+^ from the ER to the mitochondria. Ca^2+^ release, in turn, induces mitochondrial Ca^2+^ loading, with a consequent release of mitochondrial proteins involved in the apoptotic process, such as cytochrome c, the apoptosis initiating factor AIF, and the complex of second mitochondria-derived activator of caspases (SMAC) with the direct IAP binding protein with low pI (DIABLO) (61, 62). Conditions that reduce ER Ca^2+^ storage, and thus Ca^2+^ release from the ER to the mitochondria, lower the probability of Ca^2+^ dependent apoptosis (62). In *Pml*^−/−^ MEFs, both ER steady-state (Ca^2+^) values and the increase of cytosolic Ca^2+^ concentration after treatment with apoptotic stimuli like H_2_O_2_ are significantly smaller than in *Pml*^+/+^ MEFs (63). PML appears to be essential for PP2A tethering to the ER localized receptor IP3R-3, favoring IP3R-3 dephosphorylation and Ca^2+^ storage. Conversely, IP3R-3 is hyper-phosphorylated in the absence of PML and this modification decreases the Ca^2+^ release from the ER and the overload of mitochondria increasing the apoptotic threshold (64).

The effect of PML overexpression is consistent with it having a function in the regulation of survival, yet it is variable and depends on cell-type and experimental conditions. Three kinds of responses are observed upon PML overexpression: cell death without the hallmarks of apoptosis, caspase3-dependent apoptosis, and senescence. PML overexpression leads to rapid cell death without the typical features of apoptosis in immortalized rat embryonic fibroblasts (65). Caspase-3 activity is not induced upon overexpression of PML in these cells, implying that PML may quickly trigger apoptosis with a caspases-3-independent mechanism. Caspase-independent apoptosis is not unprecedented and has been also observed after staurosporine treatment, serum deprivation, and overexpression of oncogenes like *c-MYC* or *E1A* (66). Caspase-3-dependent apoptosis is triggered via P53 stabilization and the regulation of mitochondrial Ca^2+^ overload as mentioned above. Finally, introduction of PML into *Pml*^−/−^ cells leads to increased P53 levels, recruitment of P53 and its acetyltransferase CREB binding protein (CBP) which mediates P53 stabilization to PML-NBs, and cellular senescence (43, 44). PML is essential for the induction of V-H-Ras-induced senescence via P53 acetylation and activation (48). Interestingly, although all isoforms can bind P53 and recruit it to PML-NBs, only the PML-IV isoform activates P53 and triggers senescence even in the absence of PML-NBs (67).

Overexpression of PML-IV also induces senescence through an RB-dependent mechanism (67, 68). Intriguingly, during the induction of senescence, PML-NBs colocalize with RB, the E2F transcription factor (69), and senescence-associated heterochromatin foci (SAHF), suggesting that heterochromatin proteins (such as HIRA, HP1, and ASF1) transit *via* NBs before the establishment of heterochromatin foci and senescence (70, 71). PML involvement in senescence induction has recently gained central stage from studies on human ubiquitin ligase *E6AP*^−/−^ B-cell lymphomas. E6AP is an E3 ubiquitin ligase shown to target PML for degradation *via* the proteasome (72). *E6AP*^−/−^ lymphomas express elevated levels of PML and PML-NBs and show a parallel increase in markers of cellular senescence, including P21, H3K9me3, and P16 (73). Equally, the expression of PML is restored by down-regulation of E6AP in B-lymphoma cells, with concurrent induction of cellular senescence, suggesting that the overriding of PML-induced senescence is essential for B-cell lymphoma progression (73).

### PML and cell-cycle restriction

Promyelocytic leukemia function is also intertwined with a second tumor suppressor called *P21WAF1/CIP1*. P21 belongs to the cyclin-dependent kinase inhibitor family and its role in cell-cycle restriction and differentiation has been widely investigated. PML is required for the transactivation of the *P21* promoter following physiological doses of all-trans-retinoic acid (ATRA) treatment. Concentrations of ATRA in the range of 10^−7^–10^−6^ M do not activate the endogenous *P21* gene in *Pml*^−/−^ bone-marrow cells. Since P21 up-regulation results in the terminal differentiation of hematopoietic cells (74), the lack of *P21* induction in *Pml*^−/−^ cells might contribute to the role of PML in controlling hematopoietic myeloid differentiation (36). Terminal differentiation is indeed a powerful tumor suppressive mechanism, which leads a cell to a post-mitotic stage. Interestingly, PML-dependent accumulation of P21 is both P53 dependent and independent. Indeed, *PML* knock-down in *P53*^−/−^ H1299 and HCT116 cells by siRNA interference causes down-regulation of P21 expression, inhibition of the γ-irradiation-induced up-regulation of *P21*, and decreased half-life of the P21 protein (75). These data provide evidence for a P53-independent functional relationship between PML and P21 in γ-irradiation-induced DNA damage responses, and identify PML as a positive post-translational regulator of P21 in *P53*^−/−^ tumor cells.

Two lines of evidence link PML and P21 activity in normal and cancer cells. Interestingly, P21 and PML are important determinants of HSC stemness, self-renewal, and quiescence (34, 76). Experiments suggest that PML and P21 functions are important to maintain self-renewal of CML stem cells and to sustain AML leukemogenesis, respectively (77). In this context, *PML* and *P21* both act as oncogenes rather than tumor suppressors. Notably, stem cells may be endowed with unique checkpoint pathways, as compared to progenitors or more differentiated cells, suggesting that cell-cycle restriction might increase their self-renewal potential (78). Importantly, P21 is also involved in DNA repair and it is recruited to UV irradiation-induced DNA damage sites where it interacts with proliferating cell nuclear antigen (PCNA), regulates the interaction of repair factors with PCNA, and protects PCNA from degradation (79).

Previous studies also indicated that PML post-transcriptionally represses several genes involved in growth promotion and cell-cycle progression, including cyclins (D1, E1, A2, B1) and c-MYC (43, 80– 82). In addition, PML can influence entry into the cell cycle by regulating the activation of the tumor-suppressor RB. Interestingly, PML binds to the phosphatase PP1, and promotes PP1-dependent dephosphorylation of RB in neural stem cells (35). In *PML*^−/−^ mice, the amount of phosphorylated RB in NPCs is increased and correlates with higher proliferation and defective exit from the cell cycle without any substantial effect on apoptosis (35).

### PML and DNA repair

An important role for PML in the response to DNA damage and repair is also suggested by the dynamic localization of several players of the DNA damage detection and repair machinery, such as the Bloom syndrome protein (BLM) and others like the ataxia telangiectasia and Rad3 related protein (ATR), the cds1 homolog kinase 2 (CHK2), and the Nijmegen Breakage Syndrome 1 (NBS1), to PML-NBs [reviewed in (52)]. Indeed, *Pml*^−/−^ MEFs show a greatly augmented frequency of sister chromatid exchange (SCE) which is a distinctive molecular feature of Bloom syndrome cell genomic instability, suggesting that the localization of BLM to PML-NB is important to ensure BLM proper function (83). Thus, PML expression is a barrier to genomic instability, contributing to restrain the effects of DNA damage and oncogenic mutations. On the other hand, DNA repair systems are also potentially harmful for the organism, particularly in cells with a chronic source of DNA damage like cancer cells.

### PML and viral infection

Promyelocytic leukemia confers resistance on RNA viruses by interacting with viral proteins and inhibiting their functions or, in a P53-dependent way, by inducing apoptosis of infected cells (84). In this way, PML expression restrains the propagation of infection and reduces the chance of possible oncogenic mutations due to viral DNA integration into the cell genome. The IFN is the best characterized inducer of PML, and all IFNs (α, β, and γ) sharply enhance mRNA and protein levels of PML, leading to a marked increase in the number and size of PML-NBs (85). Most of the studies implicating PML in anti-viral defense have been performed with the isoforms PML III, IV, or VI, whereas those implicating PML in apoptosis and senescence were performed with PML-IV isoform.

### PML and angiogenesis

Promyelocytic leukemia affects angiogenesis by negatively regulating the Akt-mTOR pathway which controls the synthesis of the hypoxia inducible factor HIF-1α, the main factor regulating cell response to hypoxia (86). Therefore, PML loss not only decreases PML tumor suppressive functions but also amplifies tumor hypoxia responses, such as angiogenesis, migration, metabolic reprograming, epithelial-to-mesenchymal transition, tumor growth, and chemoresistance, and these functions collectively generate very aggressive tumors. The HIF-1α high, Kelch-like protein KLHL20 high (PML E3 ubiquitin ligase), the peptidyl-prolyl cis-trans isomerase NIMA-interacting Pin1 high and the resulting PML low-expression profile correlate with high-grade tumors (87). PML deficiency, indeed, leads to increased neoangiogenesis and elevated expression of pro-angiogenic factors such as the vascular endothelial growth factor (VEGF) in human and mouse tumors, thus supporting growth and spreading of the disease (86).

### PML and telomere maintenance

Promyelocytic leukemia is also involved in a mechanism of telomere maintenance known as alternative lengthening of telomeres (ALT). ALT occurs in telomerase-negative cell lines and PML forms ALT associated PML bodies (APBs) (88, 89). The appearance of APBs correlates with the stabilization of telomere length in ALT cell lines, suggesting that these bodies are responsible for the preservation of telomere length in the absence of telomerase activity (88, 89). The involvement of PML as a positive regulator of telomere maintenance on one hand suggests that its function may prevent genomic instability, on the other, it may grant telomerase-negative cancer cells a way to overcome replicative senescence.

### PML and autophagy

Autophagy is a catabolic process that allows lysosomal-mediated degradation of unnecessary or dysfunctional cellular components. Autophagy functions as an adaptive mechanism, which grants cell survival under several stress conditions (e.g., starvation, hypoxia) and maintains cellular integrity by clearing of subcellular debris and regeneration of metabolic precursors (90–93). In cells growing in a nutrient-rich environment (94, 95) autophagy is negatively regulated by mTOR and AKT signaling, two factors that are often constitutively active in AML (96). In line with these observations, mice lacking the autophagy-related protein 7 (Atg7) in HSCs develop an atypical myeloproliferative disorder, reminiscent of myelodisplastic syndrome (MDS), which progresses to AML (97). PML inhibits the AKT-mTOR signaling pathway, thus suggesting an involvement in the regulation of autophagy. A study from Laane and colleagues (98) shows that impairment of autophagy by siRNA-mediated repression of the autophagy regulator BECN1 interferes with glucocorticoid dexamethasone-mediated lymphoblastic leukemia cell death. Dexamethasone treatment leads to PML up-regulation, its interaction with AKT, and PML-dependent AKT dephosphorylation, which was required for dexamethasone-dependent cytotoxicity effects on thymocytes. Conversely, autophagy was shown to contribute to ATRA mediated differentiation and PML-RARA degradation in NB4 cells, where PML-RARA is targeted to lysosomes by the ubiquitin binding adaptor protein p62/SQSTM1 (99, 100). These studies coherently associate PML activity with autophagy induction and tumor suppression in acute promyelocytic leukemia. However, the role of autophagy in tumorigenesis and tumor maintenance is still controversial.

In conclusion, PML functions are complex, and a comprehensive model which clearly summarizes how they are interlinked is still missing. Definitely, PML mediates apoptosis and senescence, two very important strategies for limiting tumor development and progression. However, some of PML activities, such as its control of cell-cycle progression via P21 and RB or its role in telomere maintenance, hide an oncogenic potential that some tumors might exploit to survive. Thus, PML functions cannot be considered univocally tumor suppressive.

## Discussion

Promyelocytic leukemia has an important role in the regulation of stress response in normal cells. Its complex regulatory network allows integration of signals coming from several pathways. As a consequence, PML seems to be a key factor in cell fate decisions, i.e., self-renewal vs. differentiation, adaptation vs. apoptosis or senescence. *In vitro*, PML activity is necessary for the induction of apoptosis in response to many different stimuli, and overexpression of PML leads to apoptosis or senescence, as demonstrated from both *in vitro* experiments and *in vivo* observations. Despite this centrality in mediating apoptosis or cell-cycle arrest, PML inactivation is not sufficient to increase the rate of spontaneous cancers in mice. However, in murine experimental models of cancer, PML loss can cooperate with some oncogenic lesions to increase tumor initiation and progression.

**Figure 1:**
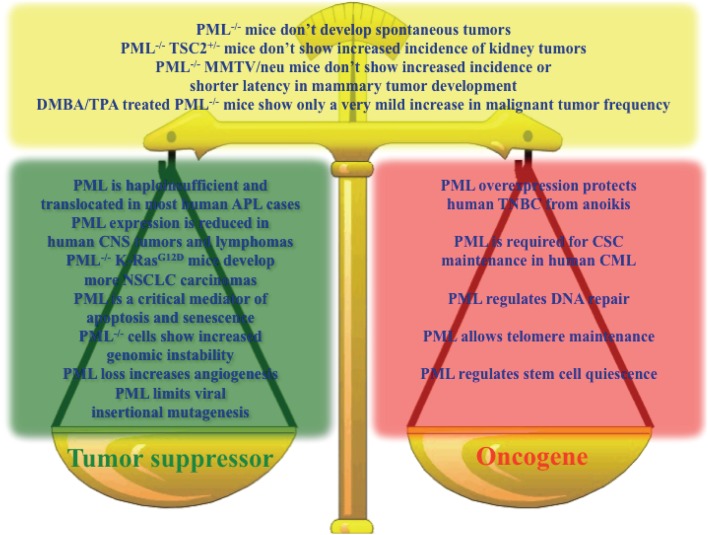
**Key results sustaining a role for PML as a tumor suppressor (green box), oncogene (red box), or in contrast with the hypothesis that PML is a tumor suppressor (yellow box)**.

Altogether, the emerging picture is that of a context-dependent contribution of PML to both tumorigenesis and tumor suppression, and it is not possible to conclude which way the balance tilts (Figure 1).

Non-small cell lung carcinoma tumorigenesis driven by the oncogene *K-Ras^G12D^*, for example, benefits from PML loss, which leads to an increased number of lung adenocarcinomas. However, *Pml*^−/−^ mice treated with the DMBA-TPA protocol, while showing increased papilloma incidence, develop few more malignant tumors, and *Tsc2*^±^ and MMTV neu driven kidney and mammary tumors, respectively, do not show differences in tumor initiation. Moreover, PML expression is required to maintain self-renewal and avoid cell death by anoikis in CML and TNBC, respectively, suggesting that some tumors may even rely on PML expression to sustain the malignant phenotype. Hence, the simple notion that PML is a general tumor suppressor that serves as a strong pro-apoptotic and pro-senescence determinant is not supported by the available data. Which of the multiple functions of PML is crucial or contrasts cancer development and maintenance depends probably on the specific physiological state of each neoplasia, and it is determined by the mutations occurring in that specific cell. Consistent with this hypothesis is the observation that PML expression correlates with high frequency of mutated P53 in TNBC. It is tempting to speculate that also the reciprocal may be true, so that reduced PML expression could affect tumors where the P53 or RB pathways are not inactivated. Furthermore, PML loss of expression in human tumors, which is mostly regulated at the level of protein stability, may be the result of the constitutive activation of common growth factor cues, which impinge into the same ERK2/PIN1/KLHL20/PML axis leading to PML protein degradation.

In recent years supporting evidence for the CSC or hierarchical model has been collected, shedding light on the crucial role of CSCs in tumor maintenance. The emerging concept that CSCs are the real biological reservoir of malignancies and that it is possible to exhaust CSC self-renewal by driving CSCs out of quiescence is a powerful paradigm which has just begun to be exploited experimentally and in clinical trials. Proof of concept experiments on this issue have been performed in murine models of CML (34) and AML (77). In this regard, PML is an interesting potential target since it is highly expressed in stem cells, restrains cell-cycle progression and maintains stem cell self-renewal [reviewed in (101)].

Future experiments should probably reassess the expression level of PML more specifically in the CSC compartment of human tumors. It is indeed possible that PML expression is confined to the CSCs and absent in the more differentiated cancer progenitors. Since CSCs very often constitute only a minor fraction of the whole tumor population, PML expression may have gone unnoticed in previous analyses, underestimating the importance of PML in the maintenance of a specific tumor.

If this is the case, PML function in support of self-renewal may be more important than its tumor-suppressor activities and the applicability of well-established therapeutic agents, like As_2_O_3_, which lead to PML degradation may be much broader than expected.

## Conflict of Interest Statement

The authors declare that the research was conducted in the absence of any commercial or financial relationships that could be construed as a potential conflict of interest.
